# 2-Phenylquinazolinones as dual-activity tankyrase-kinase inhibitors

**DOI:** 10.1038/s41598-018-19872-3

**Published:** 2018-01-26

**Authors:** Yves Nkizinkiko, Jenny Desantis, Jarkko Koivunen, Teemu Haikarainen, Sudarshan Murthy, Luca Sancineto, Serena Massari, Federica Ianni, Ezeogo Obaji, Maria I. Loza, Taina Pihlajaniemi, Jose Brea, Oriana Tabarrini, Lari Lehtiö

**Affiliations:** 1 Faculty of Biochemistry and Molecular Medicine & Biocenter Oulu, University of Oulu, Oulu, Finland; 2 Department of Pharmaceutical Sciences, University of Perugia, 06123 Perugia, Italy; 3 Biofarma research group, Centro de Investigación CIMUS, University of Santiago de Compostela, Santiago de Compostela, Spain; 4 Present Address: Center of molecular and macromolecules studies, Polish academy of science, Sienkiewicza 112, 90-363 Lodz, Poland

## Abstract

Tankyrases (TNKSs) are enzymes specialized in catalyzing poly-ADP-ribosylation of target proteins. Several studies have validated TNKSs as anti-cancer drug targets due to their regulatory role in Wnt/β-catenin pathway. Recently a lot of effort has been put into developing more potent and selective TNKS inhibitors and optimizing them towards anti-cancer agents. We noticed that some 2-phenylquinazolinones (2-PQs) reported as CDK9 inhibitors were similar to previously published TNKS inhibitors. In this study, we profiled this series of 2-PQs against TNKS and selected kinases that are involved in the Wnt/β-catenin pathway. We found that they were much more potent TNKS inhibitors than they were CDK9/kinase inhibitors. We evaluated the compound selectivity to tankyrases over the ARTD enzyme family and solved co-crystal structures of the compounds with TNKS2. Comparative structure-based studies of the catalytic domain of TNKS2 with selected CDK9 inhibitors and docking studies of the inhibitors with two kinases (CDK9 and Akt) revealed important structural features, which could explain the selectivity of the compounds towards either tankyrases or kinases. We also discovered a compound, which was able to inhibit tankyrases, CDK9 and Akt kinases with equal µM potency.

## Introduction

Tankyrases are enzymes (EC 2.4.2.30), which belong to a family of proteins known as human Diphtheria toxin-like ADP-ribosyltransferases. Two homologous tankyrases, TNKS1/ARTD5/PARP5a and TNKS2/ARTD6/PARP5b, catalyze a post-translational modification, poly-ADP-ribosylation, of target proteins. Tankyrases are very similar and both contain a catalytic ARTD domain, a sterile alpha motif (SAM) required for oligomerization and five ankyrin repeat clusters (ARCs) recognizing target proteins^1^. Tankyrases have overlapping functions in cellular signaling pathways and function e.g. in telomere homeostasis^2–5^, in elongation of centrioles and the formation of mitotic spindle during mitosis^6^, and in exocytosis of trans-Golgi vesicles containing IRAP and GLUT4^4,7,8^. Recent studies have indicated tankyrases as anti-cancer drug targets as they are involved in regulating the Wnt/β-catenin pathway known to promote the survival of cancer cells^9–11^. In essence, the inhibition of TNKSs leads to a phosphorylation of β-catenin by a destruction complex formed by Axin, adenomatous polyposis coli (APC), glycogen synthase kinase 3 β (GSK3β), and Casein kinase 1 (CK1)^12,13^. The subsequent proteasomal degradation of the phosphorylated β-catenin inhibits transcription of Wnt target genes. Tankyrases control β-catenin phosphorylation through regulation of the stability of destruction complex by ADP-ribosylating Axin and therefore inhibition of tankyrases offers an attractive strategy for treating cancers with increased Wnt-signaling^14^.

**Figure 1:**
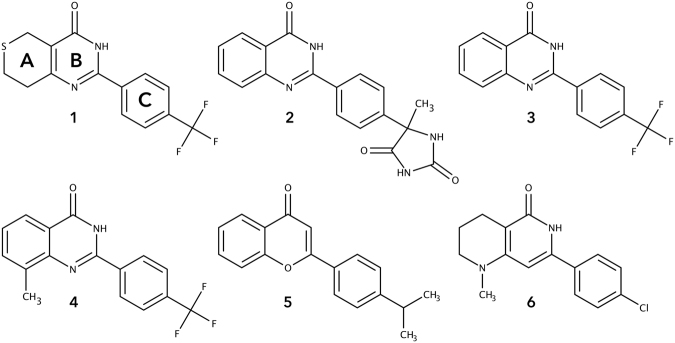
Examples of nicotinamide site binding tankyrase inhibitors.

Recently, multiple studies have reported development of potent and in some cases selective small molecule tankyrase inhibitors targeting the catalytic domain^15^. Crystal structures of catalytic domain of both tankyrases are available^16,17^, which has facilitated structure-activity relationship (SAR) studies. Tankyrase inhibitors interact with the NAD^+^ binding groove of the catalytic domain and they bind either to the nicotinamide subsite, the adenosine subsite or span across both sites^15^. The first tankyrase inhibitor XAV939 (compound **1**, Fig. 1) provided initial evidence of the involvement of tankyrase in cancerous Wnt signaling^14^. Multiple potent scaffolds resembling **1** in shape have been reported, including 2-phenylquinazolinones (2-PQ_S_) (such as compounds **2**–**4**)^18,19^, flavones (such as compound **5**)^20,21^, arylnaphthyridinones (such as compound **6**)^22^ (Fig. 1), and others such as 3-arylisoquinolin-1-ones^23^, tetrahydroquinazolin-4-ones^24^, pyrrolopyrimidinones^25^, and arylpyridopyrimidinones^22^. Here, we aimed at studying the 2-PQ class of TNKS inhibitors by synthesizing new analogues as well as assaying a large series of differently functionalized 2-PQ_S_ previously reported as Cyclin-dependent kinase 9 (CDK9) inhibitors^26^ and developed to interfere with HIV-1 Tat-mediated transcription^27,28^. We show that these compounds are in general more potent TNKS than CDK9 inhibitors and we rationalize the SAR towards tankyrases with a set of co-crystal structures.

As there was an evident potential for off targets within the kinase family for the 2-PQ compounds, we also evaluated this liability. It has been pointed out in the literature that simultaneous inhibition of certain kinases in combination with other enzymes could be used to potentiate the effect of kinase inhibitors offering a possibility for the development of double agents. For instance, it has been shown that the combination of telomerase inhibitors with tankyrase inhibitors enhances telomere shortening, leading to cell death^29^. Furthermore, synergistic effects were observed when inhibitors of telomere-regulating kinases, such as extracellular signal-related kinase 8 (ERK8), were used in combination with tankyrase inhibitors^30^. We profiled the best tankyrase-inhibiting 2-PQs against a panel of tankyrase and Wnt-related kinases and most of the analogs tested were significantly more potent against tankyrases than kinases, while one analog, compound **42**, showed similar inhibition of TNKS2 and CDK9 as well as Akt kinase. These results identified potential routes for development of double agents, but also guide the design of analogs by revealing structure-based off-target liabilities.

## Results

### SAR and structural studies

In a previous study we identified the hydantoin derivative **2** with nanomolar potency against tankyrases, which, however, was not potent in a cell-based assay (IC_50_ ≈ 1 µM)^18^. The compound was also not selective towards tankyrases, which we rationalized to be the result of polar substituent in the *para*-position of the C-ring. The low clogD of the compound (0.36) was intriguing taken into account that typically potent tankyrase inhibitors are rather hydrophobic and therefore the compound had a potential for further development. The original compound **2** was purchased commercially as a racemic mixture, and we decided to synthesize the pure (−)-enantiomer of compound **2** (hereafter named (−)**-7**) and (+)-enantiomer of compound **2** (hereafter named (+)**-8**) to evaluate the effect of the chirality (Supplementary Information).

**Figure 2:**
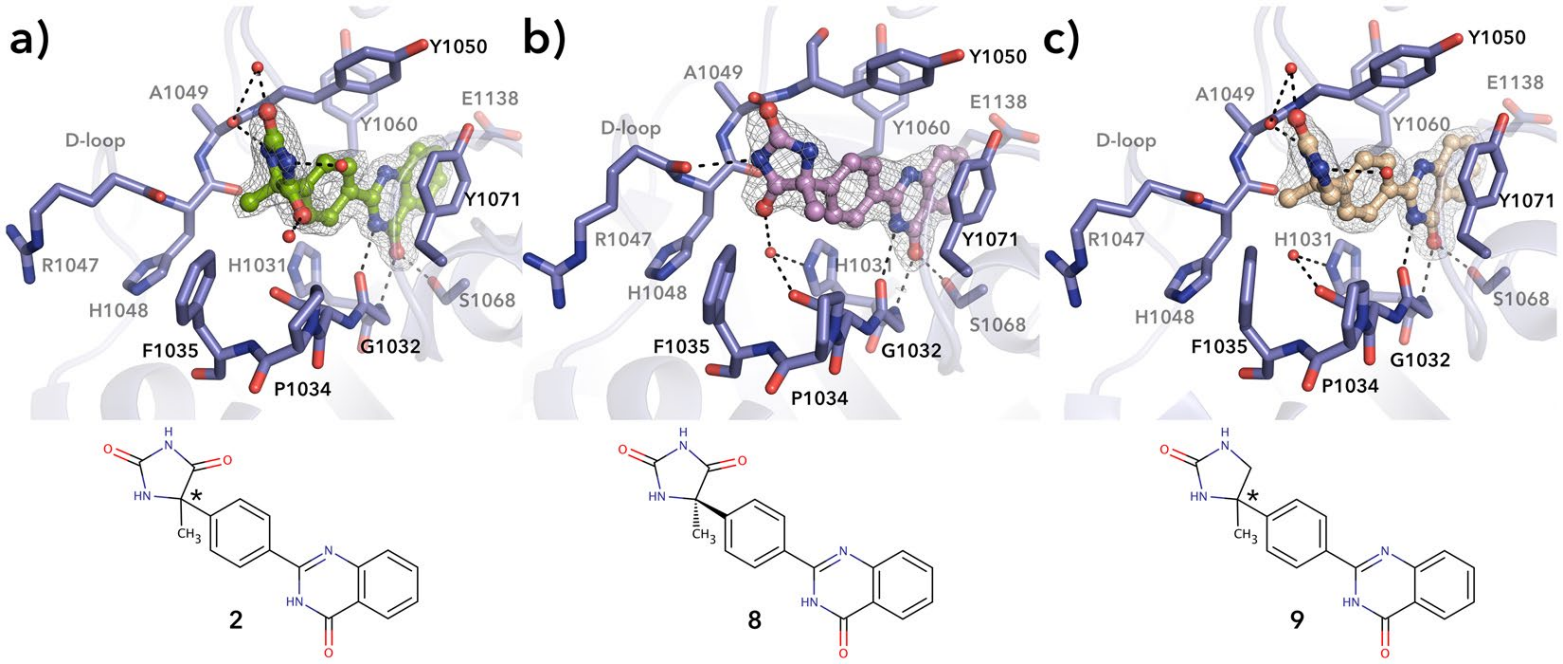
Crystal structures showing the binding mode of (**a**) **2** (PDB code 4BUY)^18^, (**b**) (+)**-8** and (**c**) **9** to TNKS2 catalytic domain. Black dashed lines represent hydrogen bonds and red spheres represent water molecules. Sigma A weighted 2Fo – Fc electron density map around the ligands is contoured at 1.5 σ.

In the TNKS2 complex structure with the commercial racemic mixture of **2** (Fig. 2a), we only observed the (−) enantiomer bound^18^. The other enantiomer, (+)**-8** was also crystallized with TNKS2 (Fig. 2b) and similarly to (−)-enantiomer, the hydantoin moiety makes one hydrogen bond with the protein *via* an amide. However, instead of the main chain carbonyl of Ala1049, the (+)-enantiomer is hydrogen bonded to the main chain carbonyl of Arg1047. When evaluated for their ability to inhibit tankyrases (−)**-7** and (+)**-8** showed a similar potency against TNKS2 (IC_50_ = 2.4 and 6.7 nM, respectively) in agreement with that of the racemic mixture **2** (IC_50_ = 3.3 nM). It is likely that the crystal environment selects for the (−)-enantiomer. Because of modest cellular potency of compound **2** (68% inhibition at 2 µM), a small modification of the hydantoin substituent was attempted to improve the cellular potency while maintaining the inhibitory activity. Removal of one of the carbonyls led to **9** with a similar binding mode as compound **2** (Fig. 2c). Again only (−)-enantiomer was observed in the co-crystal of the racemic compound. Compound **9** showed lower potency against both TNKS1 and TNKS2 (IC_50_ = 10 nM) with respect to **2** (IC_50_ = 5.9 and 3.3 nM, respectively) (Table S1) but we did not observe improved potency in the Wnt-reporter assay (72% inhibition at 2 µM; Table S1). The modification however made the compound more selective towards the tankyrases (Table S1) likely due to the removal of interactions with the α-helical regulatory domains of ARTD1 and 2, which is not present in tankyrases (Figure S1)^18^.

**Figure 3:**
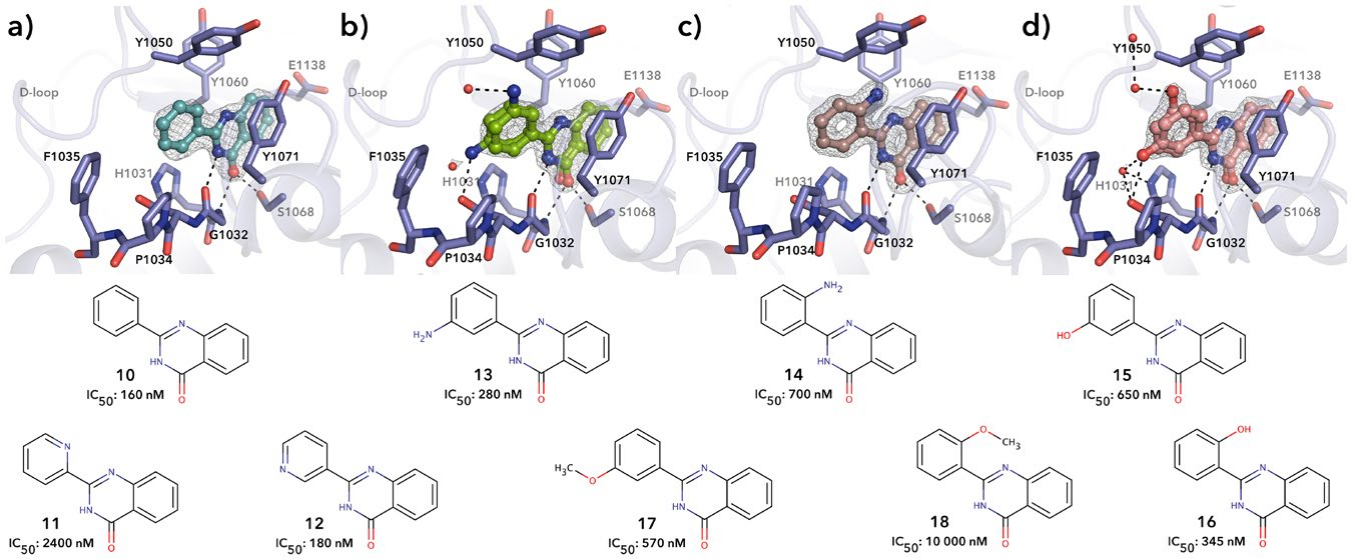
Crystal structures showing the binding mode of (**a**) **10** (PDB code 4BU3)^18^, (**b**) **13**, (c) **14**, and (d) **15** to TNKS2 catalytic domain. The C-ring contains either the amino, hydroxyl or methoxy group in *ortho-* or *meta*-position. The compounds which have a *meta*-substitution are found in double conformations in the crystal structures. Black dashed lines represent hydrogen bonds and red spheres represent water molecules. Sigma A weighted 2Fo – Fc electron density map around the ligands is contoured at 1.5 σ.

As previously reported, the basic 2-PQ scaffold **10** binds to the nicotinamide site of TNKS2 and makes typical hydrogen bonds to the active site glycine and serine residues as well as a stacking interaction with a tyrosine (Fig. 3a)^18^. In order to improve the compound we first studied the effect of the addition of hydrogen bond donors and acceptors to the *ortho*- and *meta*-positions of the phenyl ring (C-ring), which could form additional interactions with the protein (Fig. 3). Replacement of the phenyl group with a pyridine (found in **11** and **12**) could provide a hydrogen bond acceptor and would enhance the solubility of the compound. The nitrogen in the *ortho*-position however caused a drop in the potency from 160 nM to 2.4 µM likely due to lost hydrophobic interactions, while nitrogen in the *meta*-position did not change the potency. We hypothesized that the nitrogen would not be close enough to form interactions and therefore we tested amine (**13** and **14**), hydroxyl (**15** and **16**) and methoxy groups (**17** and **18**) in both positions, which could act as hydrogen bond donor, donor as well as an acceptor, and an acceptor, respectively. All the substitutions were tolerated except methoxy in *ortho*-position, which likely causes a conformational change in the compound not compatible with the binding site. Crystal structures revealed that the amine in *ortho*-position (**14**) forms a dipole-dipole interaction with the side chain of Tyr1050, which however does not seem provide additional overall binding energy (Fig. 3c). In case of substituents in the *meta*-position (**13** and **15**) the compound forms additional water mediated interactions with the protein backbone, but the compounds are in two conformations in the crystal structures indicating that the binding poses would be of similar energy and the contribution of this hydrogen bond is not providing an affinity increase (Fig. 3b and d).

**Figure 4:**
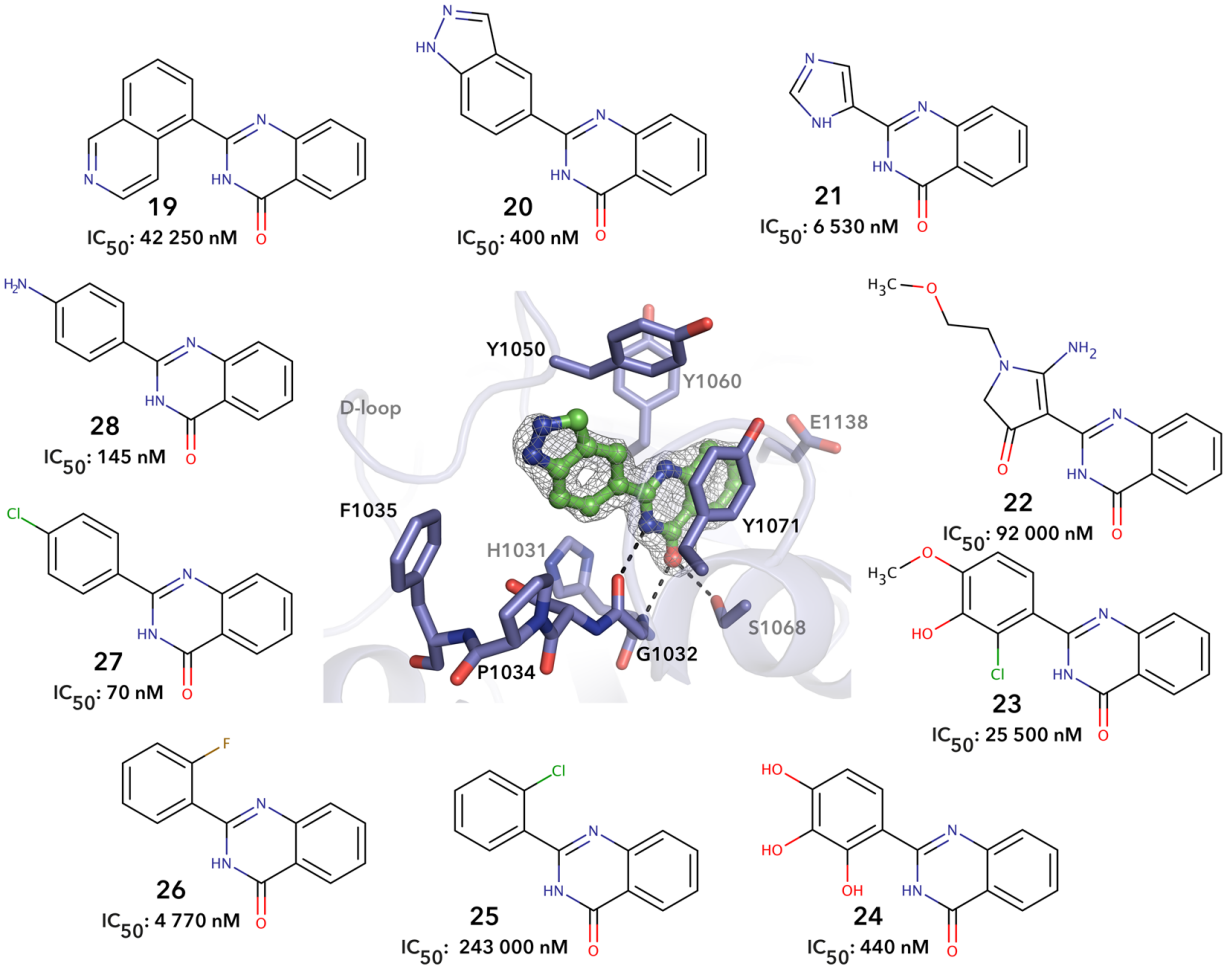
Schematic representation of **20** binding to the catalytic domain of TNKS2 as seen in the crystal structure. **20** showed higher potency against TNKS2 in comparison to *ortho-* and *meta*-substituted compounds. Black dashed lines represent hydrogen bonds and red spheres represent water molecules. Sigma A weighted 2Fo – Fc electron density map around the ligand is contoured at 1.5 σ.

Some of the 2-PQs studied had a two ring-moiety instead of the phenyl group as a C-ring. The results demonstrated that the isoquinoline ring of **19** is too large to be accommodated in the tankyrase binding pocket, while the indazolyl of **20** is well tolerated. The crystal structure reveals that the compound extends out of the narrow binding pocket of the TNKS2 (Fig. 4).

A replacement of the C-phenyl ring with a 5-membered ring in **21** was not tolerated and also highly substituted **22** showed poor inhibition (Fig. 4). Multiple substituents in the phenyl group gave contrasting results: a significant decrease in potency was observed for **23**, while multiple hydroxyl groups in **24** are tolerated like in the flavone series^20,21^. The chlorine in *ortho*-position of **25** and **26** is likely to rotate the phenyl group out of the plane as we have reported earlier for similar methyl substitution in *ortho*-position of dihydroquinazoline and pyridopyrimidinone scaffold^24^. Also the fluorine in the *ortho*-position of **27** reduced the potency despite the small size of the substituent. On the other hand, when the chlorine was placed at the *para*-position of **28**, a recovery in potency was observed with an IC_50_ of 70 nM.

**Figure 5:**
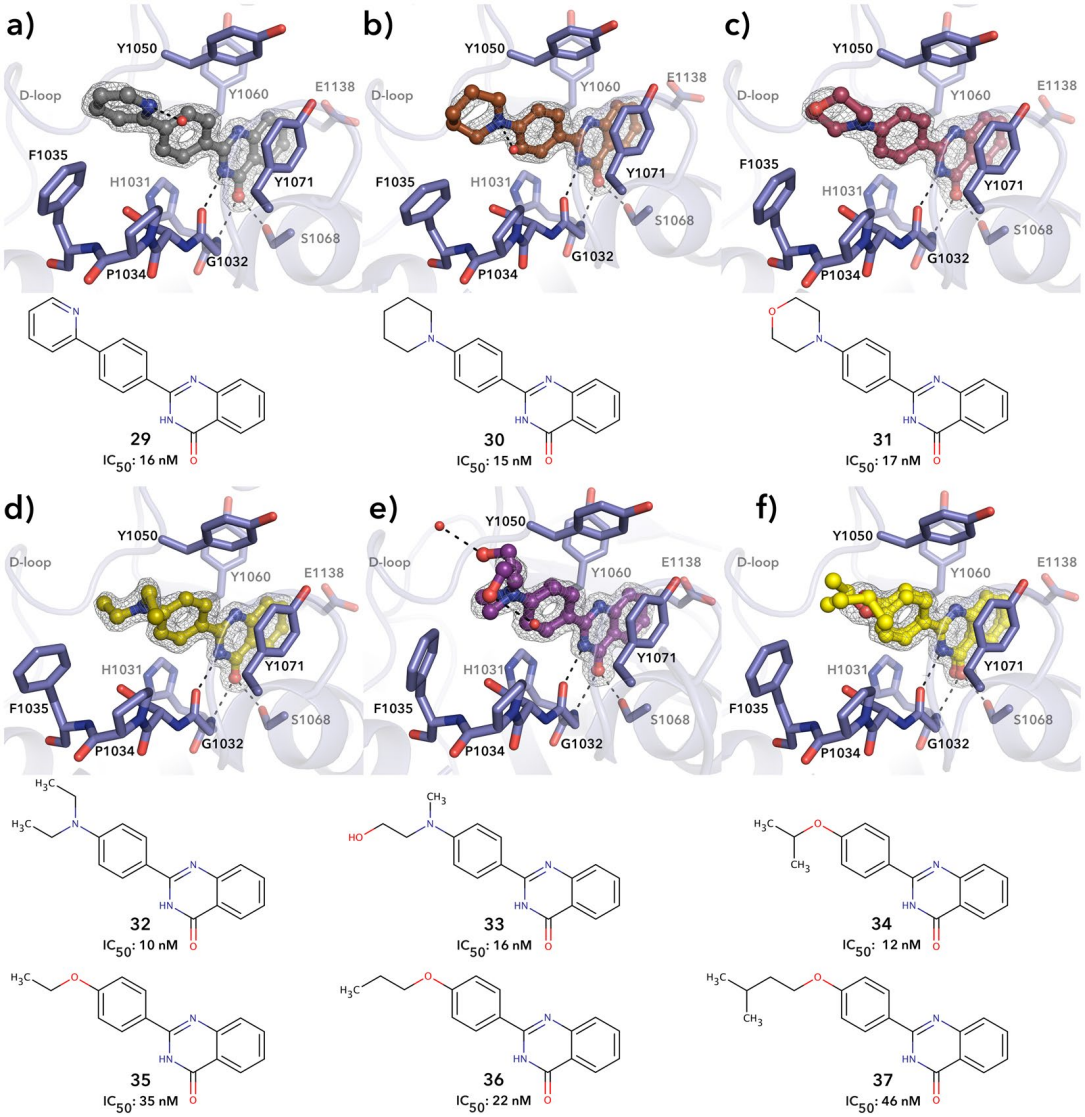
Crystal structure showing the binding mode of (**a**) **29**, (**b**) **30**, (**c**) **31**, (**d**) **32**, (**e**) **33**, (**f**) **34** to TNKS2 catalytic domain. Black dashed lines represent hydrogen bonds and red spheres represent water molecules. Sigma A weighted 2Fo – Fc electron density map around the ligands is contoured at 1.5 σ. Ile1075 was left out for clarity.

Substituents at the *para*-position of the phenyl group has been reported many times as a way to increase the potency of tankyrase inhibitors, and especially the addition of hydrophobic groups is known to improve specificity towards tankyrases^18,19,21,31^. The same trend was observed here. In particular, increasing the size of the *para*-substituent improved the potency in compounds **29**, **30**, and **31**, all characterized by an additional ring, as well as in compounds **32**, **33**, and **34**, having a branched side chain (Fig. 5). This increase in potency is caused by the hydrophobic interactions between the *para*-substituent and the side chain of Phe1035, Tyr1050 and Ile1075. A decrease in potency was however observed for compounds **35**, **36**, and **37** bearing a more linear side chain as *para*-substituent.

**Figure 6:**
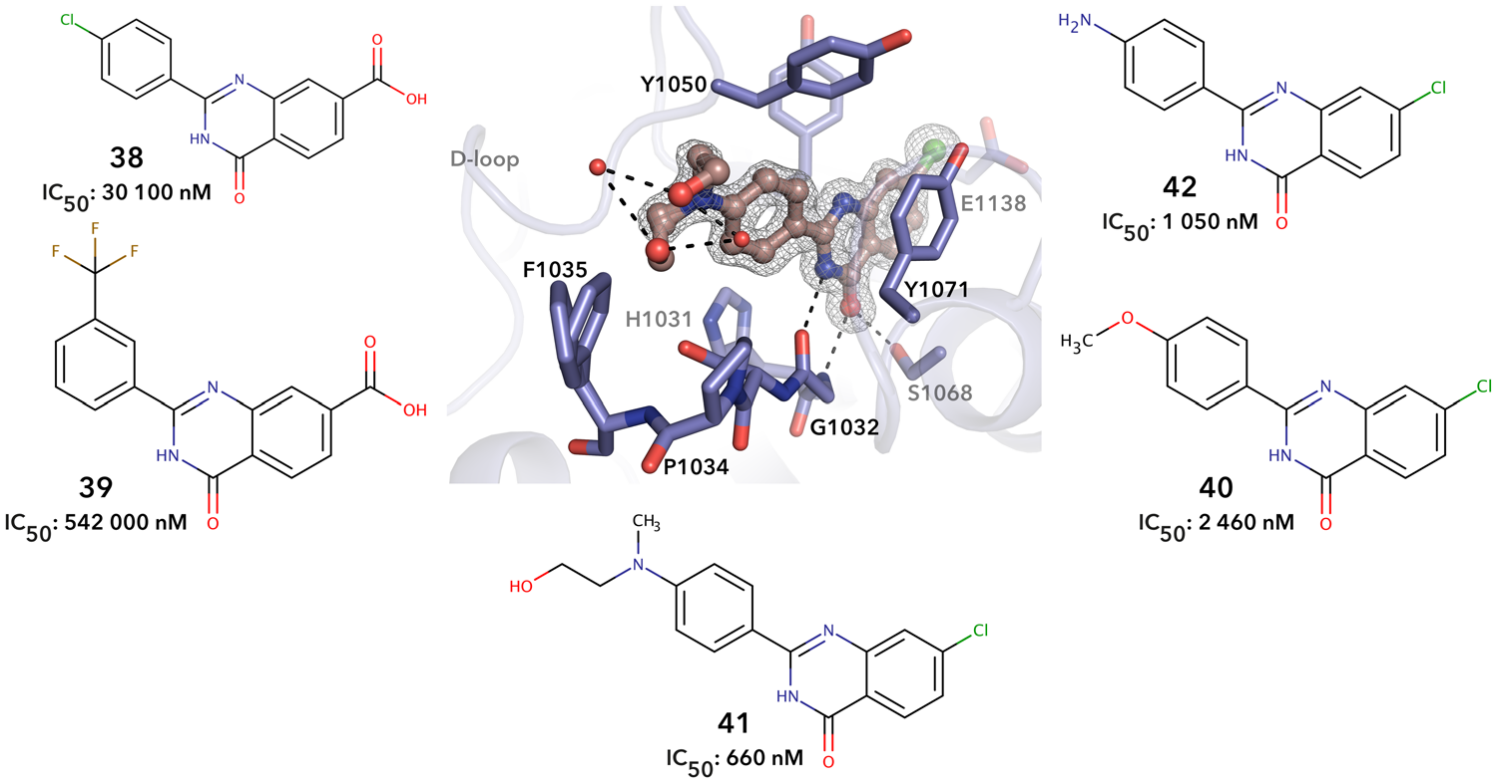
Crystal structure showing **41**. Black dashed lines represent hydrogen bonds and red spheres represent water molecules. Sigma A weighted 2Fo – Fc electron density map around the ligand is contoured at 1.5 σ.

Chlorine and carboxyl substituents in the A-ring are not compatible with the TNKS2 inhibition (Fig. 6). However, while the carboxyl derivatives, **38** and **39** cause an unfavorable interaction with the active site glutamate, the chlorine substituent of compounds **40–42** fits to the binding pocket (Fig. 6). Notably, a chlorine atom in the A ring improved CDK9 inhibition^26^.

### Wnt signaling

In order to evaluate the potential of the best tankyrase inhibitors in cells, compounds **28–37** were tested in commercial TCF/LEF reporter cell line to explore their effect on Wnt/β-catening signaling. Out of 10 tested compounds, only **30** and **32** were active at 1 µM (>50% inhibition of Wnt/β-catening signaling, Table S2). In particular, **30** showed a sub-micromolar IC_50_ value of 570 nM, whereas **32** displayed a dual behavior: at 1 µM it showed 63% inhibition but at lower concentrations, it activated Wnt/β-catening signaling (Figure S2). **33** showed only a residual activity in the assay (20% inhibition).

### Profiling of compounds against ARTDs

Compounds **30**, **32** and **33**, which were active in the reporter assay were profiled against a panel of human ARTDs to establish selectivity towards tankyrases (Table S2). Compounds **30** and **32** showed similar potencies against TNKS1/2 (30 nM/15 nM and 11 nM/10 nM, respectively), while **33** was four times more potent against TNKS2 than TNKS1 (16 nM/77 nM). Moreover, compounds **30** and **32** emerged as highly selective TNKS inhibitors, while **33** showed also inhibition of other enzymes of the family with potencies at low µM range (Table S2). Good selectivity profile coupled with the submicromolar activity in the reporter assay makes **30** the most promising tankyrase inhibitor among the studied 2-PQ_S_.

### Kinase profiling

In order to elucidate the kinase vs. tankyrase inhibition of the 2-PQs, we profiled a selection of compounds against a panel of tankyrase and Wnt-signaling related kinases. We initially utilized compounds **30** and **32**, because these compounds were more effective in cell-based assays, but we also included **20** and **42**, which were previously reported to be active against CDK9, and the known tankyrase inhibitor XAV939 **1**. Compound G007-LK, which binds to a different location in the NAD^+^ binding groove, was also studied in parallel^32^.

Results of the profiling (Tables 1, S3 and S4) showed that the best tankyrase inhibitor identified, compound **30**, did not significantly inhibit the tested kinases although it showed modest inhibition at 10 µM concentration. This is similar to **20**, which contains a bulkier two-ring substituent. Control tankyrase inhibitors **1** and G007-LK did not inhibit significantly the kinases either. Compound **32**, which has a more flexibility at the *para*-substituent modestly inhibits most tested kinases at 10 µM. Compound **42** inhibited all the tested kinases except CK2 and the potencies against CDK9 and Akt kinases are at the same level as for TNKS2 (Table 1). We rationalized that this would be a result of the presence of the chlorine substituent in the quinazolinone core and therefore we tested three further compounds, its deschloro analog **28**, and compounds **41** and **33**, against CDK9 and Akt (Table 1). The results supported our hypothesis that the chlorine substituent transforms a specific tankyrase inhibitor into a double agent with micromolar potency.

**Table 1:** Kinase profiling using selected TNKS inhibitors at 10 μM. Dose response was measured for selected kinase-inhibitor pairs and inhibition-% or an IC_50_ is shown.

Compound	CDK9	Akt	p42	p38	GSK3β	CK2	TNKS2
	34%	1%	14%	3%	57%	12%	400 nM
	31%	2%	3%	3%	11%	1%	15 nM
	16%	48%	18%	20%	4%	2%	10 nM
	6.2 µM	14.7 µM	—	—	—	—	660 nM
	16.1 µM^*^	13.3 µM	—	—	—	—	16 nM
	3.4 µM	1.6 µM	51%	45%	41%	2%	1,050 nM
	28.3 µM^*^	13.8 µM	—	—	—	—	145 nM
	12%	7%	2%	3%	7%	1%	5 nM
	9%	1%	3%	3%	3%	1%	25 nM

TNKS2 IC_50_ values are shown for comparison.

^*^The compound does not completely inhibit the enzyme at concentrations used. IC_50_ value extrapolated by the analysis software might be not accurately estimated.

### Binding modes of 41 and 42 into CDK9 and Akt

**Figure 7:**
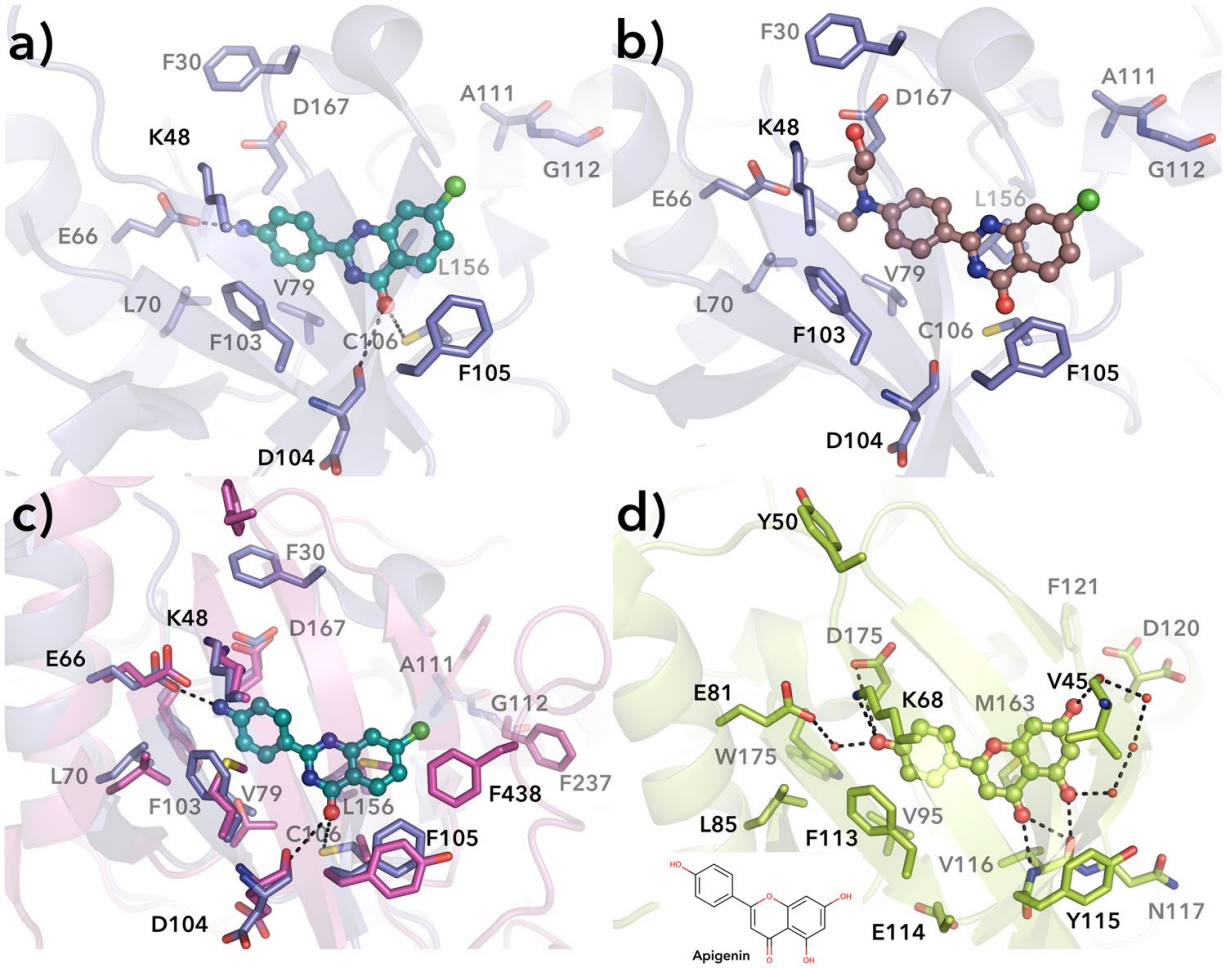
Structural representation showing the binding site of the inhibitors. (**a**) **42** and (**b**) **41** were docked into the active site of CDK9 (PDB code 3TNH). (**c**) Superposed structures of CDK9 (PDB code 3TNH, blue) and Akt (PDB code 4EKK, magenta)^41,42^ including compound **42**. (**d**) Crystal structure of CK2 complexed with apigenin (PDB code 4DGM, green)^43^. Black dashed lines represent hydrogen bonds and red spheres represent water molecules.

In order to determine structural features that make compounds **41** and **42** potent kinase/TNKS inhibitors, computational studies were performed. Docking of **42** into CDK9 structure (PDB code 3TNH) indicated potential binding modes of the compound to the kinase active site (Figure S3). In the best poses, compound **42** makes two hydrogen bonds with the side chain of Cys106 and the backbone amide of Asp104, while another hydrogen bond is formed between the *para*-amino group and the side chain of Glu66. In addition, the C-ring is well positioned for π-π stacking interaction with Phe103 (Fig. 7a). These interactions anchor **42** into the active site of CDK9 and may explain its high binding affinity in comparison to other compounds of the same series. The substitution of the *para*-amino group by a larger 2-(methylamino)ethanol ones in derivative **41** appears to abolish all of the interactions of **42** and the compound is moved out from the pocket towards Phe105 in poses similar to compound **42** (Fig. 7b, Figure S3). The loss of some interactions respect to amino derivative **42** is in line with the observed two-fold reduction in potency from 3.4 µM of **42** to 6.2 µM of **41** (Table 1).

The superposition of CDK9 (3TNH) and Akt (4EKK) structures revealed striking resemblance of the active sites with key residues being mostly conserved. Indeed, compounds **41** and **42** could maintain the same binding poses within the Akt active site as shown in Fig. 7c for compound **42**. While compound **42** is a potent Akt inhibitor (1.6 µM), in the same order of CDK9 inhibition, compound **41** is a weaker inhibitor (14.7 µM). The difference could result from a smaller binding pocket in Akt lined by Phe438.

The binding mode of the compounds described here is similar to the one observed for apigenin. Apigenin has a similar shape as 2-PQs and a co-crystal structure with CK2 (PDB code 4DGM) (Fig. 7d) resembles the binding mode predicted for CDK9. However, we did not observe inhibition of CK2 by the 2-PQs (Table 1), which could be due to the unfavorable/missing interaction with Val45 (Fig. 7d). Compound potencies, co-crystal structures, and docking to the active sites of kinases indicate that the *para*-substitution is required for improving tankyrase potency and selectivity, whereas smaller substituents in the same position also fit into the binding pocket of CDK9, CK2 and Akt. In addition, a chlorine substituent in the A-ring is beneficial for the kinase inhibitory potency but leads to a potency decrease in TNKS inhibition.

## Discussion

In this study we screened and profiled a family of CDK9 inhibitors against TNKS2. Even though most of these compounds were developed as CDK9 inhibitors, they had a quinazolinone-based scaffold, which is also a potent TNKS1/2 inhibitor scaffold. The compounds were able to inhibit TNKS2 with greater potency than CDK9. Furthermore, the SAR observed for these compounds were similar to other TNKS1/2 inhibitors that bind to the nicotinamide subsite such as flavones, arylquinazolinones, pyridopyrimidinones, dihydroquinazolinones and tetrahydroquinazolinones^18–21,24^. The most potent compounds were those characterized by a substitution in the *para*-position of the C-ring. Structural analyses revealed that the *para*-substituent improves the potency through hydrophobic interactions with Pro1034 and Phe1035 residues in the binding pocket of TNKS2. When larger *para*-substituents are present, new hydrogen bonds are created in addition to the described hydrophobic interactions, as exemplified by compounds **2** and **9**.

Profiling of the most potent tankyrase inhibitors of the series against other ARTDs confirmed the selectivity of the scaffold in general towards tankyrases. Among the most potent TNKS inhibitors, compounds **30** and **32** were also able to interfere with the Wnt/β-catenin pathway in cells, when assayed at 1 μM in the TCF/LEF reporter assay.

Notable structural features emerged for selective TNKS inhibition and dual TNKS/kinases inhibition from the profiling a set of compounds against a panel of Wnt-signaling related kinases and CDK9. The presence of a chlorine atom on the A-ring imparted the same inhibitor potency towards TNKS and CDK9 as in compounds **41** and **42**. The most potent CDK9 inhibitor of the series, compound **42** (IC_50_ = 3.4 µM), characterized by a small *para*-substituent, exhibited the same high potency also against Akt (IC_50_ = 1.6 µM). Interestingly, the anti-kinase activities are in the same order as the TNKS2 inhibition (IC_50_ = 1.1 µM) making compound **42** a promising dual agent. Thus, in order to achieve selectivity towards TNKS1/2 the *para*-substitution of the C-ring of the 2-PQs is crucial and A-ring substitution shifts the potency towards kinases. The study gives potential routes for the optimization of the 2-PQs scaffold to obtain selective TNKS inhibitors while avoiding kinase inhibition, and/or to generate more potent double agents in the future.

Even though the idea of develop double agent for intentional co-inhibition of kinases and TNKSs is fairly new, there are several examples where ARTD inhibitors were discovered to inhibit other therapeutic targets or potentiate the synergistic effect of other drugs. The typical and most studied example is that of Olaparib, which was discovered as an ARTD1 inhibitor and later found to inhibit other ARTDs including TNKSs^31^. Olaparib (AZD2281) is currently marketed under the trade name of Lynparza and it was approved by FDA for the treatment of homologous recombination (HR)-deficient cancers having BRCA1/2 mutations^33^. Co-inhibition of ARTDs and histone deacetylases (HDAC) with Olaparib and Suberoylanilide Hydroxamic Acid (SAHA) respectively is an example where a positive synergy was demonstrated using HR-proficient ovarian cancer cell lines against which Olaparib alone has minimal efficacy as a single agent^34^. The same combination was also proven to be effective in triple negative breast cancer cells and in a xenograft mouse model^35^. In another scenario, TNKS inhibitor XAV939 was shown to reduce ICP0- and ERK-dependent Herpes simplex viral replication^36^. Furthermore, TNKS inhibitors enhanced the inhibitory effect of Erlotinib, which is an EGFR inhibitor, by blocking the YAP signaling pathway^37^. This is a classical example where dual inhibition of two important pathways, YAP and Wnt/β-catenin, was achieved by the use of TNKS inhibitors. Considering that most TNKS inhibitors like **1** are widely used in cell-based assays and in different contexts, the possibility of an off-target effect should be taken into account by e.g. testing in parallel multiple compounds that are now available. On the other hand by careful crafting of the structure it could be possible to develop agents with dual activity on a single compound like in the case of **42**.

## Methods

### Protein expression and purification

Human ARTD1–3 were expressed as full length proteins^18,24^ whereas ARTD4-7, ARTD10 and ARTD12 were expressed as protein fragments containing the catalytic domain and they were purified according to the reported protocols^18,24,38,39^.

### Activity assay

The activity assay used to measure the potency of the compounds against TNKS2 is based on the quantification of NAD^+^^38^. The reactions were carried out in 96-well plate (Greiner bio-one, U-shaped) and the conditions were the same as reported earlier (Table S5)^24^.

### Screening of compounds and potency measurements

The compounds were stored in 100% DMSO at −20 °C. All the compounds were diluted in the TNKS2 assay buffer (50 mM Bis-Tris Propane, 0.5 mM TCEP, 0.01% Triton-100x). Control reactions were used to exclude the effect of compound fluorescence and quenching from the calculations. One dose-response curve was measured using half-log dilutions and in quadruplicates for all the compounds and for the most potent compounds the measurement was repeated three times and on separate days. The pIC_50_ values were calculated and fitted with nonlinear regression using 4-parameters with GraphPad Prism (version 5.0 for windows).

### Profiling of the compounds against ARTDs

The compounds showing high potency against TNKS2 were profiled against other human ARTDs (Table S2). The assay conditions used in the profiling were previously optimized for each enzyme (Table S5)^24,40^. For all compounds a dose-response curve was measured using half-log dilutions. The concentration of NAD^+^ was kept constant (500 nM) during the potency measurements.

### Kinase profiling

The kinase functional assays were performed using a combination of kinase and 1.5 μM peptide substrate in the presence of ATP. The reaction mixture was analyzed on the Caliper LabChip 3,000 (Caliper, Hopkinton, MA) by the electrophoretic mobility shift of the fluorescent substrate and phosphorylated product. For the inhibition of human CK2α-1, the experiments were carried out with ADP-Glo kinase Assay Kit (Promega V9,101). Assays are described in detail in the Supplementary Methods and Table S6.

### Reporter assay

The Wnt Signaling Pathway TCF/LEF Reporter HEK293 Cell Line (BPS Bioscience, catalog #60,501) was used to study the effect of the most potent tankyrase inhibitors on Wnt signaling. The Wnt assay was performed using a procedure modified from the BPS Bioscience protocol. Details are given in the Supplementary Methods.

### Crystallization, data collection, refinement and docking

Crystallization of the catalytic domain of TNKS2 (residues 952–1,161) was done similarly as previously described^20^. Details are given in the Supplementary Methods and Table S7.

### Chemistry

The majority of the 2-PQ_S_ herein reported were synthesized as previously described^26^, while compounds **10–12** and **22**, **27**, **38**, **39** were purchased from vendors (**10–11**, Mir Biotech; **12**, Life Chemicals; **22**, BAS; **27**, Vitas M Lab; **38** and **39**, Enamine). Purity of the acquired compounds was measured by HPLC and were higher than 95%. Although compound **2** was commercially available as racemate its synthesis was never been reported before and is shown in (Scheme S1), together with the synthesis of its enantiomers (−)-**7** and (+)-**8** (Scheme S2), as well as compound **9** (Scheme S3).

## Electronic supplementary material

Supplementary Information
