# Supplementary material for: 2-Phenylquinazolinones as dual-activity tankyrase-kinase inhibitors
Source: Sci Rep. 2018 Jan 26;8:1680. doi: 10.1038/s41598-018-19872-3 (PMC5785997; doi:10.1038/s41598-018-19872-3)
Supplement: Supplementary file 1 — Supplementary Information [file 41598_2018_19872_MOESM1_ESM.pdf]

Supplementary information

2-Phenylquinazolinones as dual-activity tankyrase-kinase inhibitors

Yves Nkizinkiko[1], Jenny Desantis[2,¤], Jarkko Koivunen[1,¤], Teemu Haikarainen[1,¤], Sudarshan Murthy[1], Luca Sancineto[2], Serena Massari[2], Federica Ianni[2], Ezeogo Obaji[1], Maria I. Loza[3], Taina Pihlajaniemi[1], Jose Brea[3], Oriana Tabarrini[2,*] & Lari Lehtiö[1,*]

[1]Faculty of Biochemistry and Molecular Medicine & Biocenter Oulu, University of Oulu, Oulu, Finland

[2]Department of Pharmaceutical Sciences, University of Perugia, 06123 Perugia, Italy.

[3]Biofarma research group, Centro de Investigación CIMUS, University of Santiago de Compostela, Santiago de Compostela, Spain

CONTENTS

Supplementary methods 2

Kinase profiling 2

Reporter assay 2

Crystallization, data collection, refinement and docking 3

Supplementary Table S1. Profiling of 2 and 9 and inhibition of Wnt-reporter in cell assay. 4

Supplementary Table S2. Profiling of the most potent inhibitors and Wnt/β-catening signaling. 5

Supplementary Table S4. Kinase profiling using selected TNKS inhibitors at 10 μM 6

Supplementary Table S5. Conditions used in the activity assay for each ARTD enzyme 7

Supplementary Table S6. Assay conditions of the evaluated kinases 7

Supplementary Table S7. Data collection and refinement statistics 8

Supplementary Table S7. contd. 9

Supplementary Figure S1. Superposition of TNKS2-2 complex and ARTD1 structure 10

Supplementary Figure S2. Reporter assay dose-response curves for compounds 30 and 32. 10

Supplementary Figure S3. Structural representation predicted binding poses 11

Chemical synthesis 12

The synthesis of compounds 2, 7-9 and for enantioseparation analyses 12

General 14

General procedure for racemic resolution 14

Enantioselective high-performance liquid chromatography analyses 18

References 19

Supplementary methods

Kinase profiling

The kinase functional assays were performed in 384-wells plates using a 30 µl reaction volume. The reactions were run in reaction buffer (**Table S6**). The reaction was initiated by a combination of kinase and 1.5 µM peptide substrate in the presence of ATP. The reaction mixture was analyzed on the Caliper LabChip 3,000 (Caliper, Hopkinton, MA) by the electrophoretic mobility shift of the fluorescent substrate and phosphorylated product. Inhibition data were calculated by comparison to no enzyme control reactions for 100% inhibition and DMSO only reactions for 0% inhibition. Dose-response curves were generated to determine the concentration required to inhibit 50% of kinase activity (IC_{50}). Staurosporine (Invitrogen BP2,451) was included as a positive control of each enzymatic assay. The profiling of the kinases was done at 1 and at 10 µM of the compound. For the inhibition of human CK2α-1, the experiments were carried out in a white 384-microplate low flange (Corning 3,572) with ADP-Glo kinase Assay Kit (Promega V9101) and CK2α-1 Kinase Enzyme System (Promega V4,482). The test compounds and standard staurosporine (Invitrogen BP2451), 25 ng/well CK2α-1 kinase and 50 µM ATP were added in a final volume of 10 µl/well using reaction buffer supplied by kit as assay buffer. The reaction mixture was incubated in gentle shaking for 60 minutes at room temperature. After the incubation 10 µl of ADP-Glo reagent was added and incubated in gentle shaking for 40 minutes at room temperature. Finally 20 µl of Kinase Detection Reagent was added and the plate was incubated on a shaker for 30 minutes at room temperature. Luminescence (1,000 ms) was measured with Perkin Elmer EnSpire Multimode plate reader.

Reporter assay

The Wnt Signaling Pathway TCF/LEF Reporter HEK293 Cell Line (BPS Bioscience, catalog #60501) was used to study the effect of the most potent tankyrase inhibitors on the Wnt signaling. Reporter HEK293 cells were grown at 37ºC with 5% CO_2 using Dulbecco's Modified Eagle's Medium (DMEM, Sigma), supplemented with 10% of fetal bovine serum (Lonza), 1% non-essential amino acid (Sigma), 1X of penicillin and streptomycin (Sigma), and 400 µg/ml of Geneticin (Roche). Wnt-3a and L cells (ATCC) were cultured in Dulbecco’s modified Eagle’s medium supplemented with 10% fetal bovine serum, penicillin and streptomycin at 37ºC in a 5% CO_2. Wnt3a and control conditioned medium preparation has been described previously[1]. The Wnt assay was performed using a procedure modified from the BPS Bioscience protocol. 30,000 HEK293 cells/well in 40 µl of serum-free DMEM were plated on 96-well plate (white wells, clear

bottom, PerkinElmer). 10 µl of 50 mM LiCl in serum-free DMEM with 5x concentration of DMSO or tankyrase inhibitors was added. After 16 hours of incubation, 25 µl of Wnt3a or control conditioned medium supplemented with DMSO or tankyrase inhibitors was added on cells and cells were incubated for additional 24 hours. After the second incubation, the 96-well plates were balanced in room temperature for 30 minutes. Wnt3a induced luciferase activity was determined using ONE-Glo Luciferase Assay System (Promega) according to manufacturer's protocol.

Crystallization, data collection, refinement and docking

Crystallization of the catalytic domain of TNKS2 (residues 952-1,161) was done similarly as previously described.[2] The compounds were soaked into the crystals by immersing the crystals into a soaking solution containing 0.1 M Tris (pH 8.5), 250 mM NaCl, 22% of PEG3,350, 0.2 M lithium sulphate and 100 µM of the compound. After five days of the soaking, the crystals were submerged into the same soaking solution supplemented with 20% of glycerol and thereafter flash cooled in liquid nitrogen. The data were collected at ESRF (Grenoble, France) on beamline ID23-1 and at the Diamond Light Source (Didcot, UK) at beamline I02, I03 and I04-1. The processing of the data were done with XDS[3] (**Table S7**). Structures were determined by molecular replacement with MOLREP[4] using TNKS2 structure complexed with nicotinamide (3U9H) as a template or with Difference Fourier method deriving the starting phases from 3U9H. The refinement of the structures was done with REFMAC5.[5,6] Final manual building, analysis and visualization of the structures were made with Coot[7] and PyMOL (www.pymol.org). All chemical structures were drawn with Marvin (Marvin 5.7.0 2011, ChemAxon, http://www.chemaxon.com). The docking studies were performed using GOLD software.[8]

Supplementary Table S1. Profiling of **2** and **9** against tankyrases and human ARTD1/2 and inhibition of Wnt-reporter in cell assay.

	**2** (nM)	**9** (nM)
TNKS1	5.9	10
TNKS2	3.3	10
ARTD1/PARP1	374	1 000
ARTD2/PARP2	340	870
Wnt (Inh.%, 2 µM)	68 %	72 %

Supplementary Table S2. Summary of the profiling of the most potent TNKS2 inhibitors against a panel of human ARTDs and Wnt/β-catening signaling.

	**30**	**32**	**33**
Wnt (HEK293) (1 µM)	61 % (IC_{50} 570 nM)	63 %	20 %
ARTD5/TNKS1	30 nM	11 nM	77 nM
ARTD6/TNKS2	15 nM	10 nM	16 nM
ARTD1/PARP1	18,000 nM	30,000 nM	1,700 nM
ARTD2/PARP2	8,600 nM	24,000 nM	860 nM
ARTD3/PARP3	23,000 nM	2,100 nM	3,800 nM
ARTD4/PARP4	12,000 nM	5,500 nM	2,000 nM
ARTD7/PARP15	>10,000 nM*	>10,000 nM*	>10,000 nM*
ARTD8/PARP14	>10,000 nM*	>10,000 nM	3,200 nM
ARTD10/PARP10	>10,000 nM*	>10,000 nM*	3,100 nM
ARTD12/PARP12	>10,000 nM*	>10,000 nM*	>10,000 nM*
ARTD15/PARP16	>10,000 nM*	>10,000 nM*	>10,000 nM*

* No inhibition at the highest concentration used (10,000 nM).

Supplementary Table S3. Kinase profiling using selected TNKS inhibitors at 1 μM

**Compound** (TNKS2 IC_{50})	**AKT** (% Inhib.)	**CDK9** (% Inhib.)	**ERK2** (% Inhib.)	**GSK3β** (% Inhib.)	**p38α** (% Inhib.)	**CK2** (% Inhib.)
**20** (400 nM)	1 ± 1	7 ± 4	3 ± 1	14 ± 1	1 ± 1	4 ± 3
**30** (15 nM)	1 ± 1	12 ± 4	2 ± 1	1 ± 1	3 ± 1	1 ± 1
**32** (10 nM)	1 ± 1	13 ± 4	2 ± 1	1 ± 1	1 ± 1	2 ± 1
**42** (1,050 nM)	-36 ± 2	17 ± 5	14 ± 1	4 ± 1	5 ± 1	1 ± 1
**1** (5 nM)	1 ± 1	11 ± 3	2 ± 2	1 ± 1	2 ± 2	2 ± 1
**G007-LK** (25 nM)	2 ± 1	8 ± 3	2 ± 1	1 ± 1	1 ± 1	1 ± 1

Supplementary Table S4. Kinase profiling using selected TNKS inhibitors at 10 μM

**Compound** (TNKS2 IC_{50})	**AKT** (% Inhib.)	**CDK9** (% Inhib.)	**ERK2** (% Inhib.)	**GSK3β** (% Inhib.)	**p38α** (% Inhib.)	**CK2** (% Inhib.)
**20** (400 nM)	1 ± 1	34 ± 4	14 ± 2	57 ± 1	3 ± 2	12 ± 4
**30** (15 nM)	2 ± 2	31 ± 4	3 ± 1	11 ± 3	3 ± 3	1 ± 1
**32** (10 nM)	48 ± 4	16 ± 3	18 ± 3	4 ± 4	20 ± 2	2 ± 1
**42** (1,050 nM)	89 ± 1	78 ± 2	51 ± 1	41 ±1	45 ± 1	2 ± 2
**1** (5 nM)	7 ± 4	12 ± 1	2 ± 1	7 ± 1	3 ± 2	1 ± 1
**G007-LK** (25 nM)	1 ± 1	9 ± 1	3 ± 1	3 ± 1	3 ± 1	1 ± 1

Supplementary Table S5. Conditions used in the activity assay for each ARTD enzyme.

**Protein (nM)**	**NAD^+ (nM)**	**Incubation time (hour)**	**Buffer**
ARTD1 (5)	500	1	50 mM Tris pH 8, 20 µg/mL activated DNA and 20 mM $MgCl_2$
ARTD2 (10)	500	3	50 mM Tris pH 8, 10 µg/ml DNA and 2 mM $MgCl_2$
ARTD3 (100)	500	1	100 mM PIPES pH 7, 60 µg/ml DNA, 10 mM $MgCl_2$
ARTD4 (400)	500	2.5	50 mM Na-phosphate pH 7.5, 0.5 mM TCEP, 1 mg/mL BSA
ARTD5 (15)	500	22	50 mM Bis-Tris propane pH 7, 0.5 mM TCEP, 0.01 %Triton-x-100
ARTD6 (15)	500	21	50 mM Bis-Tris propane pH 7, 0.5 mM TCEP, 0.01 %Triton-x-100
ARTD7 (350)	250	2	50 mM Na-phosphate pH 7, 500 nM SRPK2
ARTD8 (500)	500	18	50 mM Na-phosphate pH 7.0
ARTD10 (400)	500	2.5	50 mM Tris pH 7, 2,000 µM SRPK2
ARTD12 (500)	500	20	50 mM Na-phosphate pH 7.0
ARTD15 (500)	500	24	50 mM HEPES, 2mM $NiCl_2$, pH 7.0

Supplementary Table S6. Assay conditions of the evaluated kinases

Kinase	[ATP] (µM)	Substrate	Incubation time (min)	Reaction buffer
AKT	30	5-FAM-GRPRTSSFAEG-$CONH_2$	30	100 mM HEPES (pH 7.5), 10 mM $MgCl_2$, 0.003% Brij35, 0.004% Tween 20
CDK9	20	5-FAM-RRRFRPASPLRGPPK-OH	60	100 mM HEPES (pH 7.5), 10 mM $MgCl_2$, 0.003% Brij35, 0.004% Tween 20
ERK2	25	5-FAM-IPTSPITTTYFFFKKK-COOH	45	100 mM HEPES (pH 7.5), 10 mM $MgCl_2$, 0.003% Brij35, 0.004% Tween 20
GSK-3β	4	5-FAM-KRREILSRRPPSYR-COOH	180	100 mM HEPES (pH 7.5), 10 mM $MgCl_2$, 0.003% Brij35, 0.004% Tween 20
P38aplha	250	5-FAM-IPTSPITTTYFFFKKK-COOH	60	100 mM HEPES (pH 7.5), 10 mM $MgCl_2$, 0.003% Brij35, 0.004% Tween 20

Supplementary Table S7. Data collection and refinement statistics for the TNKS2 crystal structures.

**Compound**	**8**	**9**	**13**	**14**	**15**	**20**	**29**
**PDB code**	**5OWT**	**5OWS**	**5NT0**	**5NWC**	**5NVC**	**5NSX**	**5NVF**
Beam line	DIAMOND IO2	ESRF ID23-1	ESRF ID30A-1	ESRF ID23-1	ESRF ID23-1	ESRF ID30A-1	ESRF ID23-1
Wavelength (Å)	0.979490	0.87260	0.96500	0.953723	0.953723	0.96500	0.953723
Space group	$C222_1$	$C222_1$	$C222_1$	$C222_1$	$C222_1$	$C222_1$	$C222_1$
Cell dimensions a, b, c (Å)	89.00, 98.23, 113.38	91.56, 98.39, 118.44	90.87, 97.61, 118.77	91.15, 98.12, 118.85	91.13, 98.38, 119.43	91.14, 97.85, 118.45	90.81, 98.54, 119.74
Resolution (Å)	30 - 2.20 (2.20-2.26)	50 - 1.80 (1.80-1.85)	45 - 1.75 (1.75-1.80)	50 - 1.50 (1.50-1.54)	45 - 1.60 (1.60-1.64)	45 - 1.80 (1.80-1.85)	45 - 1.55 (1.55-1.59)
R_{merge}	11.6(118.8)	8.4 (94.7)	6.4 (68.5)	6.8 (61.9)	6.8 (101.6)	5.9 (71.2)	6.4 (59.8)
I / σI	12.6 (1.64)	14.3 (1.91)	12.9 (2.2)	11.0 (1.92)	15.2 (2.12)	13.4 (2.0)	11.8 (2.0)
Completeness (%)	99.8 (99.9)	100 (100.0)	99.7 (98.7)	99.4 (99.3)	98.8 (97.8)	98.1 (97.3)	99.7 (99.9)
Redundancy	6.6 (6.8)	7.52 (7.83)	4.6 (4.7)	4.36 (4.05)	5.20 (5.30)	4.6 (4.8)	4.25 (4.25)
**Refinement**							
R_{work} / R_{free}	0.190/0.237	0.168/0.194	0.168/0.194	0.183/0.206	0.179/0.202	0.174/0.199	0.177/0.201
**B-factors**							
Protein	45.0	29.8	31.8	24.0	27.0	35.2	21.6
Inhibitor	41.3	24.5	22.8	18.5	20.4	30.4	17.4
**R.m.s.d.**							
Bond lengths (Å)	0.014	0.019	0.011	0.007	0.008	0.013	0.009
Bond angles (°)	1.716	1.958	1.447	1.379	1.316	1.524	1.392
**Ramachandran plot (%)**							
Favored regions	96.0	99.5	99.0	99.0	98.8	98.5	99.3
Additionally allowed regions	4.0	0.5	1.0	1.0	1.2	1.5	0.7

Supplementary Table S7 contd. Data collection and refinement statistics for the TNKS2 crystal structures.

**Compound**	**30**	**31**	**32**	**33**	**34**	**35**	**41**
**PDB code**	**5NVH**	**5NT4**	**5NWD**	**5NWB**	**5NUT**	**5NVE**	**5NWG**
Beam line	ESRF ID23-1	ESRF ID30A-1	ESRF ID23-1	ESRF ID23-1	ESRF ID23-1	ESRF ID23-1	ESRF ID23-1
Wavelength (Å)	0.953723	0.96500	0.953723	0.953723	0.97930	0.953723	0.953723
Space group	$C222_1$	$C222_1$	$C222_1$	$C222_1$	$C222_1$	$C222_1$	$C222_1$
Cell dimensions a, b, c (Å)	90.80, 98.20, 119.3	90.09, 98.15, 118.38	90.85, 98.34, 118.78	90.00, 98.20, 119.1	91.00, 98.10, 118.1	90.75, 98.40, 118.99	90.69, 98.40, 119.93
Resolution (Å)	50 - 1.60 (1.60 -1.65)	49 - 1.90 (1.90-1.95)	45 - 1.45 (1.45-1.49)	45 - 1.60 (1.60-1.64)	45 - 1.60 (1.60-1.64)	45 - 1.50 (1.50-1.55)	45 - 1.40 (1.40-1.44)
R_{merge}	7.5 (72.6)	11.6 (113.0)	4.4 (76.7)	8.0 (71.5)	5.8 (89.0)	7.7 (83.0)	9.1 (52.9)
I / σI	11.3 (1.85)	11.4 (2.03)	15.4 (1.87)	11.6 (2.0)	16.2 (1.77)	10.7 (1.78)	11.6 (2.0)
Completeness (%)	99.6 (99.8)	100 (100.0)	99.7 (99.8)	99.5 (99.7)	100 (100)	99.8 (100)	99.0 (97.5)
Redundancy	5.0 (5.0)	6.69 (6.68)	5.2 (5.3)	5.2 (5.3)	6.67 (6.69)	5.20 (5.30)	3.8 (3.6)
**Refinement**							
R_{work} / R_{free}	0.179/0.200	0.175/0.204	0.178/0.192	0.181/0.205	0.176/0.198	0.179/0.196	0.143/0.169
**B-factors**							
Protein	22.0	31.3	23.8	22.4	26.9	24.1	20.5
Inhibitor	22.0	28.4	20.1	17.6	21.2	17.9	36.4
**R.m.s.d.**							
Bond lengths (Å)	0.007	0.012	0.007	0.008	0.007	0.009	0.008
Bond angles (°)	1.428	1.520	1.295	1.434	1.290	1.360	1.388
**Ramachandran plot (%)**							
Favored regions	99.0	98.8	99.5	99.3	99.0	99.0	99.3
Additionally allowed regions	1.0	1.2	0.5	0.7	1.0	1.0	0.7

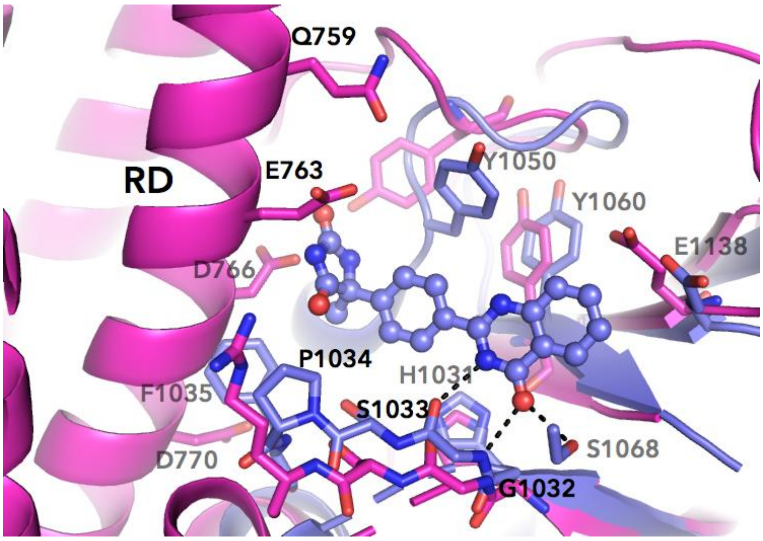

Supplementary Figure S1. Superposition of TNKS2-2 complex (blue, PDB code 4BUY) and ARTD1 structure (magenta, PDB code 4UND[10]) showing the potential interacting residues of the regulatory domain (RD) of ARTD1 and the compound.

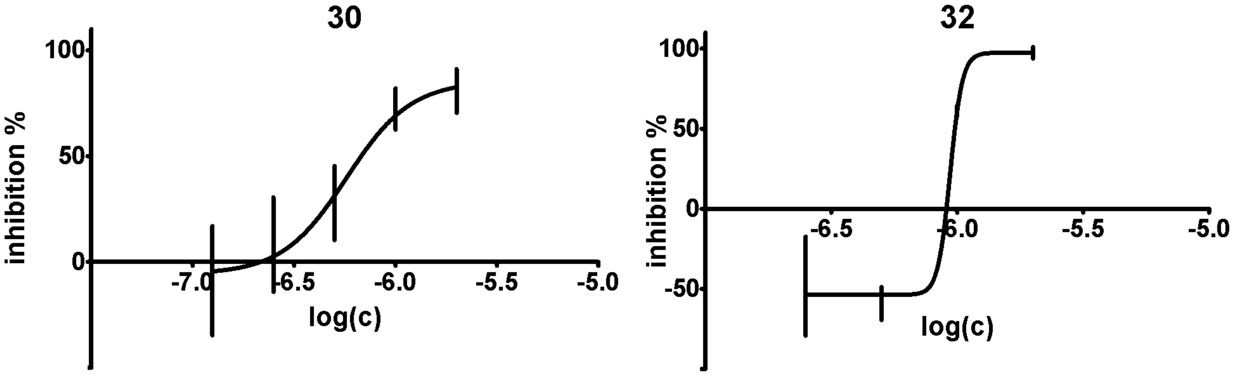

Supplementary Figure S2. Reporter assay dose-response curves for compounds **30** and **32**.

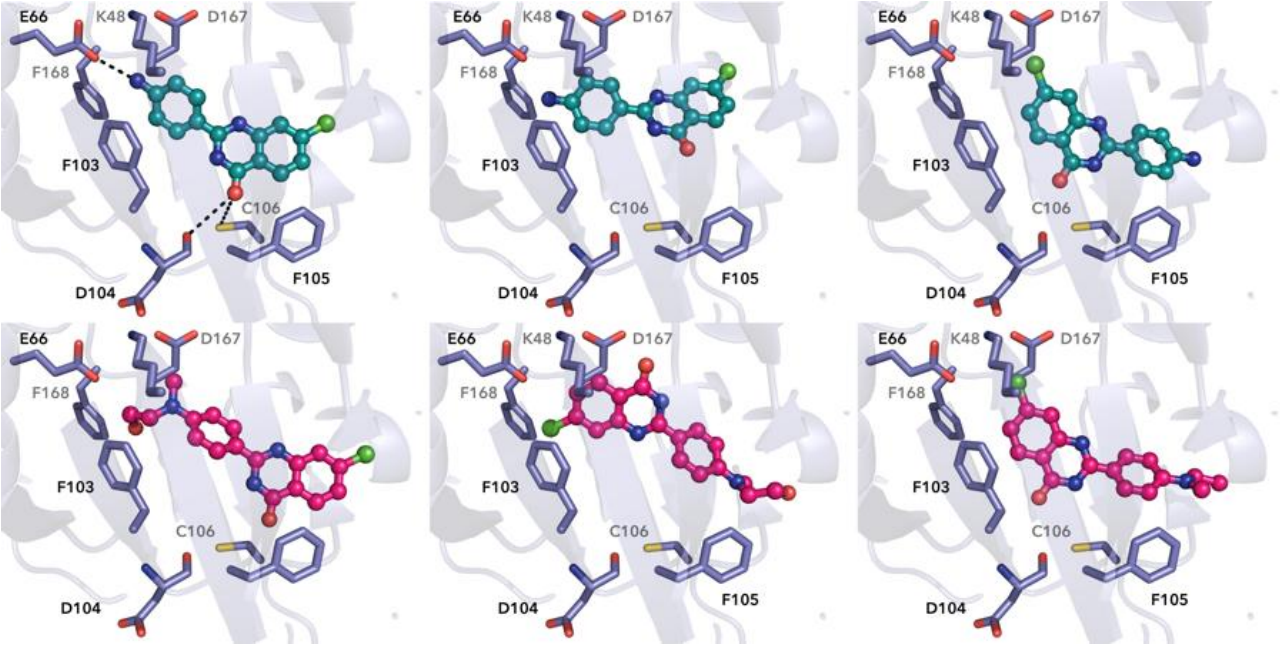

Supplementary Figure S3. Structural representation predicted binding poses of compounds to the active site of CDK9 (PDB code 3TNH[11]). a-c) Three different representative binding poses were obtained for 42 and the one shown in panel a was the highest ranked one. d- f) Three different poses were also obtained when 41 was docked into the active site of CDK9 (PDB code 3TNH).

Chemical synthesis

The synthesis of compounds 2, 7-9 and for enantioseparation analyses

Hydantoin derivative **43**[12] was synthesized as racemic mixture by reacting 1-(*p*-tolyl)ethanone, potassium cyanide, ammonia, and carbon dioxide (conveniently generated from ammonium bicarbonate) in EtOH by means of the classical four-components Bucherer–Bergs's reaction[13]. After oxidation in the presence of $KMnO_4$ and NaOH in H_2O, the key carboxylic acid **44**[12] was obtained. The successive reaction of acid **44** with anthranilamide in presence of the coupling reagent *N,N,N′,N′*-tetramethyl-O-(benzotriazol-1-yl)uronium tetrafluoroborate (TBTU) gave the racemate **45**. Hydantoin phenylquinazolinone derivative **2** was then obtained by cyclization in the presence of potassium *tert*-BuOK in *tert*-BuOH

Scheme S1[a]

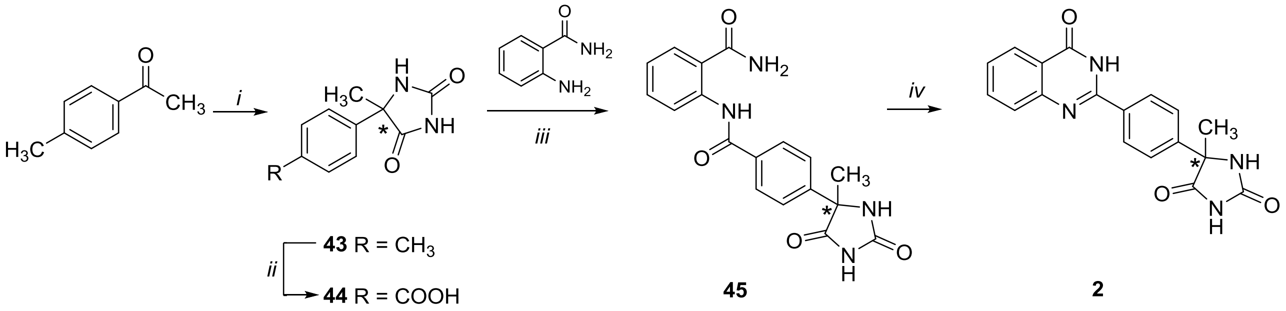

[a] Reagents and conditions: (i) KCN, NH_4HCO_3, NH_4OH, EtOH, 60°C; (ii) $KMnO_4$, NaOH, H_2O, 95°C; (iii) TBTU, DIPEA, dry DMF, rt; (iv) *tert*-BuOK, *tert*-BuOH, rt.

Using an analogous procedure, the two enantiomers **(-)-7** and **(+)-8** were prepared, as depicted in Scheme 2. The racemic resolution of the hydantoin intermediate **43** was accomplished by fractional crystallization using (-)-(S)-1-phenylethanamine and (+)-(R)-1-phenylethanamine, giving the enantiopure (e.e. % >99, as determined by HPLC) compounds (-)-**43** and **(+)-43**, which were found levorotatory and dextrorotatory, respectively, according to the literature. Then, starting from the enantiomers (-)-**43** and (+)-**43** and following the synthetic route already described, the enantiopure **(-)-7** (e.e. % 96.4) and **(+)-8** (e.e. % 93.6) were prepared through intermediates (-)-**44** and (-)-**45** and (+)-**44** and (+)-**45**, respectively.

Scheme S2[a]

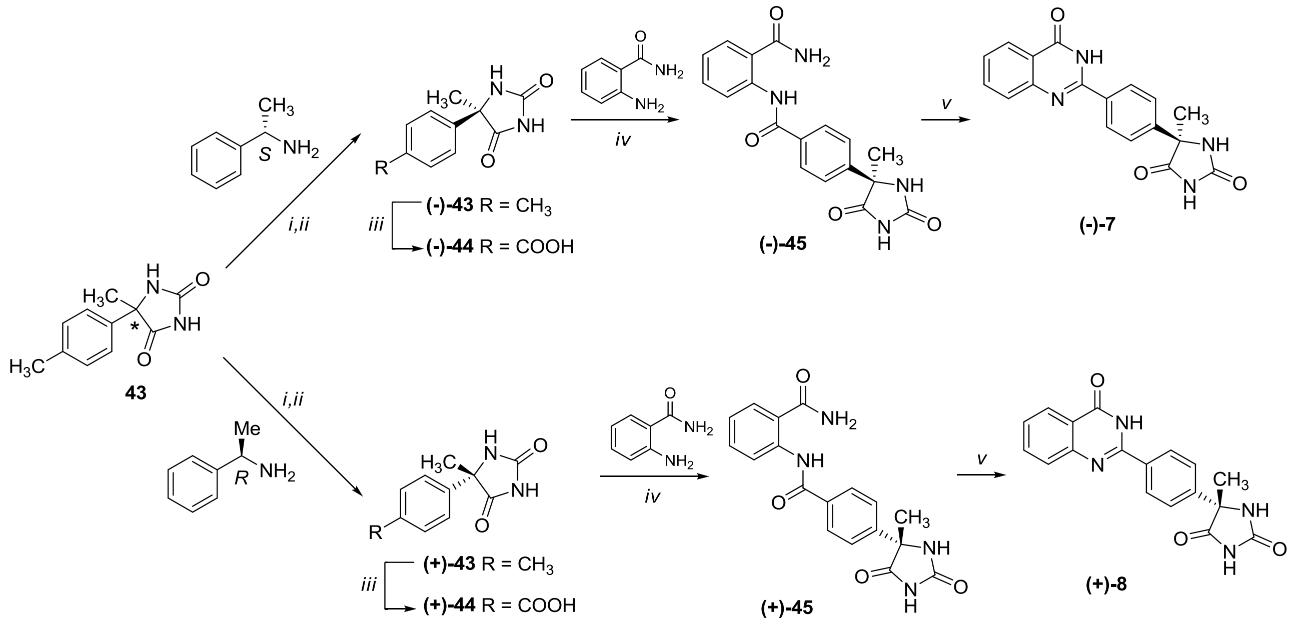

[a] Reagents and conditions: (i) NaOH, H_2O, 80° C; (ii) HCl 2N; (iii) $KMnO_4$, NaOH, H_2O, 95°C; (iv) TBTU, DIPEA, dry DMF, rt; (v) *tert*-butoxide, *tert*-butanol, rt.

The imidazolidinone derivative **9** was synthesized as racemic mixture as reported in Scheme 3. The reduction of the hydantoin ring of intermediate **43** using diisobutylaluminium hydride (DIBAL-H) led to the imidazolidin-2-one derivative **46**, whose structure was confirmed by NOE analysis. Compound **46** was then brominated by using *N*-bromosuccinimide (NBS) and azobisisobutyronitrile (AIBN) to give the benzyl bromide **47** in mixture with a 40% unreacted compound **46**. The final coupling reaction of impure compound **47** with anthranilamide, using CuCl and K_2CO_3 in DMSO, gave the target compound **9**.

Scheme S3[a]

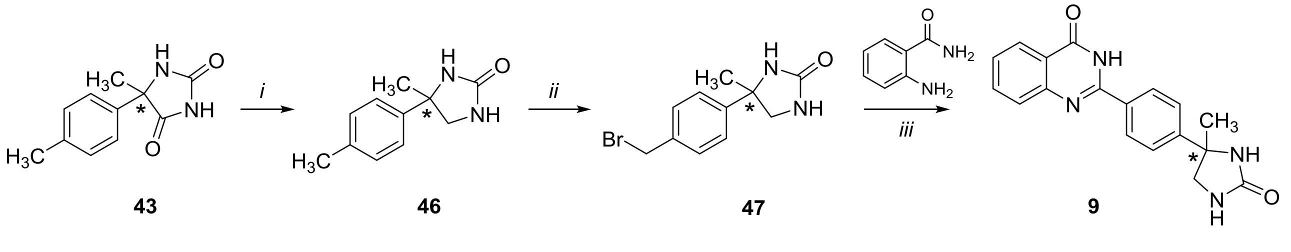

[a] Reagents and conditions: (i) DIBAL-H, dry toluene, reflux; (ii) NBS, AIBN, dry CH_3CN, reflux; (iii) CuCl, K_2CO_3, DMSO, 120 °C.

General

Commercially available starting materials, reagents, and solvents were used as supplied. All reactions were routinely checked by TLC on silica gel 60F254 (Merck) and visualized by using UV or iodine. Flash column chromatography was performed on Merck silica gel 60 (mesh 230-400). After extraction, organic solutions were dried over anhydrous Na_2SO_4, filtered, and concentrated with a Büchi rotary evaporator at reduced pressure. Yields are of purified product and were not optimized. Melting points were determined by capillary method on Büchi 9,100 electrothermal apparatus and are uncorrected. Optical rotations were measured in a 50 mm cell using the D line of sodium at 23°C as temperature. $[\alpha]_D$ values were measured in ethanol with 0.02 g of pure enantiomer in 2 mL of solution. HRMS spectra were registered on Agilent Technologies 6,540 UHD Accurate Mass Q-TOF LC/MS, HPLC 1,290 Infinity. Purity of the target compounds was determined by LC/MS on Agilent Technologies 6,550 iFUNNEL Q-TOF equipped with HPLC 1,290 Infinity with DAD detector and evaluated to be higher than 95%. HPLC conditions to assess the purity of final compounds were as follows: column, Phenomenex AERIS Widepore C4, 4.6 mm × 100 mm (6.6 μm); flow rate, 0.85 mL/min; acquisition time, 10 min; DAD 190−650 nm; oven temperature, 30 °C; gradient of acetonitrile in water containing 0.1% of formic acid (0−100% in 10 min). The enantiomeric excess of compounds was evaluated by HPLC with a Chirobiotic T column. ^{1}H NMR and ^{13}C NMR spectra, as well as 2D ^{1}H NMR NOESY, were recorded on Bruker Avance DPX-200 and Bruker Avance DRX-400MHz using residual solvents such as dimethylsulfoxide (δ = 2.48) as an internal standard. Chemical shifts were recorded in ppm (δ) and the spectral data are consistent with the assigned structures. The spin multiplicities are indicated by the symbols s (singolet), d (doublet), t (triplet), q (quartet), m (multiplet), and bs (broad singolet).

General procedure for racemic resolution

5-Methyl-5-(4-methylphenyl)imidazolidine-2,4-dione (43). The title compound was prepared as reported in the literature[12]. Compound **43**: White solid, 86% yield. ^{1}H-NMR (400 MHz, DMSO-d_6): δ = 1.50 (s, 3H, CH_3), 2.25 (s, 3H, CH_3), 7.20 (d, J = 8.0 Hz, 2H, aromatic CH), 7.30 (d, J = 8.0 Hz, 2H, aromatic CH), 8.50 and 10.55 (s, each 1H, NH) ppm. A mixture of the racemic hydantoin derivative **43** (0.5 g, 2.4 mmol), the appropriate chiral base (0.47 ml, 1.5 mmol) and NaOH (0.07 g, 0.75 mmol) in H_2O (4.8 ml) was heated up to 80-90°C in order to obtain a homogeneous solution. After cooling, crystals were allowed to grow for 4h. After filtration, the diastereoisomeric salt was decomposed with excess of 2N HCl and the precipitated of hydantoin enantiomer was filtered, washed with H_2O and dried.

(-)-(R)-5-Methyl-5-(4-methylphenyl)imidazolidine-2,4-dione [(-)-43]. The title compound was prepared by following the general procedure using (-)-(S)-1-phenylethanamine[12]. Compound **(-)-43**: White solid, yield 11%, e.e. >99% (HPLC); $[\alpha]^{23}_{D}$ -83. ^{1}H-NMR (400 MHz, DMSO-d_6): δ = 1.50 (s, 3H, CH_3), 2.25 (s, 3H, CH_3), 7.20 (d, J = 8.0 Hz, 2H, aromatic CH), 7.30 (d, J = 8.0 Hz, 2H, aromatic CH), 8.50 and 10.55 (s, each 1H, NH) ppm.

(+)-(S)-5-Methyl-5-(4-methylphenyl)imidazolidine-2,4-dione [(+)-43]. The title compound was prepared by following the general procedure using (+)-(R)-1-phenylethanamine[14]. Compound **(+)-43**: White solid, yield 11%, e.e. >99% (HPLC); $[\alpha]^{23}_{D}$ +85. ^{1}H-NMR (400 MHz, DMSO-d_6): δ = 1.50 (s, 3H, CH_3), 2.25 (s, 3H, CH_3), 7.20 (d, J = 8.0 Hz, 2H, aromatic CH), 7.30 (d, J = 8.0 Hz, 2H, aromatic CH), 8.50 and 10.55 (s, each 1H, NH) ppm.

4-(4-Methyl-2,5-dioxoimidazolidin-4-yl)benzoic acid (44). The title compound was prepared as reported in the literature[12]. Compound **44**: White solid, 63% yield. ^{1}H-NMR (200 MHz, DMSO-d_6): δ 1.60 (s, 3H, CH_3), 7.50 (d, J = 8.4 Hz, 2H, aromatic CH), 7.80 (d, J = 8.4 Hz, 2H, aromatic CH), 8.50 and 10.70 (s, each 1H, NH), 13.00 (bs, 1H, COOH) ppm.

(-)-(R)-4-(4-methyl-2,5-dioxoimidazolidin-4-yl)benzoic acid [(-)-44]. The title compound was prepared by using the same procedure employed to prepare compound **44**[12]. Compound **(-)-44**: White solid, 70% yield. ^{1}H-NMR (200 MHz, DMSO-d_6): δ 1.65 (s, 3H, CH_3), 7.55 (d, J = 8.6 Hz, 2H, aromatic CH), 7.95 (d, J = 8.6 Hz, 2H, aromatic CH), 8.80 and 10.90 (s, each 1H, NH), 13.20 (bs, 1H, COOH) ppm.

(+)-(S)-4-(4-Methyl-2,5-dioxoimidazolidin-4-yl)benzoic acid [(+)-44]. The title compound was prepared by using the same procedure employed to prepare compound **44**[12]. Compound **(+)-44**: White solid; 45% yield. ^{1}H-NMR (200 MHz, DMSO-d_6): δ 1.75 (s, 3H, CH_3), 7.55 (d, J = 8.6 Hz, 2H, aromatic CH), 7.95 (d, J = 8.6 Hz, 2H, aromatic CH), 8.70 and 10.80 (s, each 1H, NH), 13.20 (bs, 1H, COOH) ppm.

2-{[4-(4-Methyl-2,5-dioxoimidazolidin-4-yl)benzoyl]amino}benzamide (45). A mixture of compound **44** (1 g, 4.27 mmol), anthranilamide (0.58 g, 4.27 mmol), TBTU (1.37 g, 4.27 mmol), and DIPEA (3.35 ml, 19.21 mmol) in dry DMF was maintained at room temperature for 3 days. The reaction mixture was poured into ice/water, acidified (pH 6) with 2N HCl, obtaining a precipitate,

which was purified by flash chromatography eluting with $CHCl_3$/MeOH (97:3) to give **45** (0.2 g, 14%): White solid. ^{1}H-NMR (200 MHz, DMSO-d_6): δ 1.70 (s, 3H, CH_3), 7.20 (t, J = 8.5 Hz, 1H, aromatic CH), 7.50-7.70 (m, 3H, aromatic CH), 7.80 (m, 4H, aromatic CH and amide NH), 8.45 (bs, 1H, amide NH), 8.75 (m, 2H, aromatic CH and hydantoin NH), 10.80 and 13.00 (s, each 1H, hydantoin NH and amide NH) ppm.

(-)-(R)-2-{[4-(4-Methyl-2,5-dioxoimidazolidin-4-yl)benzoyl]amino}benzamide [(-)-45]. The title compound was prepared by using the same procedure used for the synthesis of compound **45**. Compound **(-)-45**: White solid, 5% yield. ^{1}H-NMR (200 MHz, DMSO-d_6): δ 1.70 (s, 3H, CH_3), 7.20 (t, J = 8.5 Hz, 1H, aromatic CH), 7.50-7.70 (m, 3H, aromatic CH), 7.80 (m, 4H, aromatic CH and amide NH), 8.45 (bs, 1H, amide NH), 8.75 (m, 2H, aromatic CH and hydantoin NH), 10.80 and 13.00 (s, each 1H, hydantoin NH and amide NH) ppm.

(+)-(S)-2-{[4-(4-Methyl-2,5-dioxoimidazolidin-4-yl)benzoyl]amino}benzamide [(+)-45]. The title compound was prepared by using the same procedure used for the synthesis of compound **45.** Compound **(+)-45**: White solid, 13% yield. ^{1}H-NMR (200 MHz, DMSO-d_6): δ 1.70 (s, 3H, CH_3), 7.20 (t, J = 8.5 Hz, 1H, aromatic CH), 7.50-7.70 (m, 3H, aromatic CH), 7.80 (m, 4H, aromatic CH and amide NH), 8.45 (bs, 1H, amide NH), 8.75 (m, 2H, aromatic CH and hydantoin NH), 10.80 (s, each 1H, hydantoin NH), 13.00 (s, each 1H, amide NH) ppm.

5-Methyl-5-{4-[(4-oxo-3,4-dihydroquinazolin-2-yl)carbonyl]phenyl}imidazolidine-2,4-dione (2). To a suspension of compound **45** (0.085 g, 0.24 mmol) in *tert*-butanol (3 ml), potassium *tert*-butoxide (0.13 g, 1.2 mmol) was added and the mixture was maintained at room temperature overnight. The solvent was evaporated in vacuum, the residue was diluted with H_2O and acidified (pH 5) with 2N HCl, obtaining a precipitate, which was purified by crystallization by EtOH/H_2O to give **2** (0.053 g, 66%): White solid, mp 318 °C. ^{1}H-NMR (400 MHz, DMSO-d_6): δ 1.65 (s, 3H, CH_3), 7.50 (t, J = 7.4 Hz, 1H, aromatic CH), 7.60 (d, J = 8.3 Hz, 2H, aromatic CH), 7.70 (d, J = 8.0 Hz, 1H, aromatic CH), 7.80 (t, J = 7.1 Hz, 1H, aromatic CH), 8.00-8.20 (m, 3H, aromatic CH), 8.70 and 10.85 (s, each 1H, hydantoin NH), 12.00 (s, each 1H, amide NH) ppm; ^{13}C-NMR (101 MHz, DMSO-d_6): δ 25.4, 64.3, 121.4, 126.0, 126.3, 127.1, 127.9, 128.3, 132.6, 135.1, 143.5, 149.1, 152.3, 156.6, 162.6, 1,77.0 ppm. HRMS (ESI): *m/z* calculated for $C_{18}H_{14}N_4O_3$ $[M+H]^+$: 335.1145, found: 335.1145.

(-)-(R)-5-Methyl-5-{4-[(4-oxo-3,4-dihydroquinazolin-2-yl)carbonyl]phenyl}imidazolidine-2,4-dione [(-)-44 (7)]. The title compound was prepared by using the same procedure used for the synthesis of compound **2**. Compound **(7**: White solid, 53% yield, mp 323 °C, e.e. >96.4% (HPLC). ^{1}H-NMR (400 MHz, DMSO-d_6): δ 1.65 (s, 3H, CH_3), 7.50 (t, *J* = 7.4 Hz, 1H, aromatic CH), 7.60 (d, *J* = 8.3 Hz, 2H, aromatic CH), 7.70 (d, *J* = 8.0 Hz, 1H, aromatic CH), 7.80 (t, *J* = 7.1 Hz, 1H, aromatic CH), 8.00-8.20 (m, 3H, aromatic CH), 8.70 and 10.85 (s, each 1H, hydantoin NH), 12.00 (s, each 1H, amide NH) ppm; ^{13}C-NMR (101 MHz, DMSO-d_6): δ 25.4, 64.3, 121.4, 126.0, 126.3, 127.1, 127.9, 128.3, 132.6, 135.1, 143.5, 149.1, 152.3, 156.6, 162.6, 177.0 ppm. HRMS (ESI): *m/z* calculated for $C_{18}H_{14}N_4O_3$ $[M+H]^+$: 335.1145, found: 335.1144.

(+)-(S)-5-Methyl-5-{4-[(4-oxo-3,4-dihydroquinazolin-2-yl)carbonyl]phenyl}imidazolidine-2,4-dione [(+)-8]. The title comound was prepared by using the same procedure used for the synthesis of compound **2**. Compound **8**: White solid, 57% yield, mp 323 °C, e.e. >96.4% (HPLC). ^{1}H-NMR (400 MHz, DMSO-d_6): δ 1.65 (s, 3H, CH_3), 7.50 (t, *J* = 7.4 Hz, 1H, aromatic CH), 7.60 (d, *J* = 8.3 Hz, 2H, aromatic CH), 7.70 (d, *J* = 8.0 Hz, 1H, aromatic CH), 7.80 (t, *J* = 7.1 Hz, 1H, aromatic CH), 8.00-8.20 (m, 3H, aromatic CH), 8.70 and 10.85 (s, each 1H, hydantoin NH), 12.00 (s, each 1H, amide NH) ppm; ^{13}C-NMR (101 MHz, DMSO-d_6): δ 25.4, 64.3, 121.4, 126.0, 126.3, 127.1, 127.9, 128.3, 132.6, 135.1, 143.5, 149.1, 152.3, 156.6, 162.6, 177.0 ppm. HRMS (ESI): *m/z* calculated for $C_{18}H_{14}N_4O_3$ $[M+H]^+$: 335.1145, found: 335.1142.

4-Methyl-4-(p-tolyl)imidazolidin-2-one (46). To a suspension of compound **1** (0.3 g, 1.46 mmol) in dry toluene (15 ml), a 1M solution of DIBAL-H in toluene (14.69 ml, 14.69 mmol) was added dropwise, the resulted mixture was maintained at reflux overnight. After cooling, the reaction mixture was quenched with MeOH, diluted with H_2O, acidified (pH 4) with 2N HCl and then extracted with EtOAc, dried over Na_2SO_4, filtered, and evaporated to dryness yielding **46** (0.22 g, 78%): White solid. ^{1}H-NMR (400 MHz, DMSO-d_6): δ 1.40 (s, 3H, CH_3), 2.20 (s, 3H, CH_3), 3.20 and 3.30 (d, *J* = 8.7 Hz, each 1H, CH_2), 6.20 and 6.85 (s, each 1H, NH), 7.10 and 7.20 (d, *J* = 8.0 Hz, each 2H, aromatic CH) ppm.

4-(4-(Bromomethyl)phenyl)-4-methylimidazolidin-2-one (47). To the solution of compound **46** (1.20 g, 6.30 mmol) in dry acetonitrile (9 ml), NBS (1.23 g, 6.93 mmol) and AIBN (0.103 g, 0.63 mmol) were added , the mixture was maintained at reflux for 5 h. The reaction mixture was diluted with CH_2Cl_2 and the organic layers washed with 2N HCl, saturated solution of $NaHCO_3$, and brine. The organic layers were dried over Na_2SO_4, filtered, and evaporated to dryness obtaining a residue

purified by flash chromatography eluting with $CHCl_3$/MeOH (98:2) to give **47** in mixture with the starting compound **46** (0.3 g, 19% yield of **47**): Light-yellow solid. ^{1}H-NMR (200 DMSO-d_6): δ 1.50 (s, 3H, CH_3), 3.20-3.30 (m, 2H, CH_2), 4.70 (s, 2H, $\mathit{CH_2}$Br), 6.25 and 7.00 (bs, each 1H, NH), 7.15 and 7.25 ppm (d, J = 8.3 Hz, each 2H, aromatic CH).

2-(4-(4-Methyl-2-oxoimidazolidin-4-yl)phenyl)quinazolin-4(3H)-one (9). A mixture of compound **47** (0.260 g, 0.96 mmol), anthranilamide (0.131 g, 0.96 mmol), CuCl (0.002 g, 0.02 mmol), and K_2CO_3 (0.133 g, 0.96 mmol) in DMSO (2 ml) was maintained open to air at 120 °C overnight. The reaction mixture was poured into ice/water obtaining a precipitate, which was purified by flash chromatography eluting with $CHCl_3$/MeOH (95:5) to give **9** (0.03 g, 9%): Yellow solid, mp 336 °C. ^{1}H-NMR (400 MHz, DMSO-d_6): δ 1.60 (s, 3H, CH_3), 3.20 and 3.50 (d, J = 8.8 Hz, each 1H, CH_2), 6.30 and 7.10 (s, each 1H, NH), 7.35-7.60 (m, 3H, aromatic CH), 7.70-7.85 (m, 2H, aromatic CH), 8.10-8.20 (m, 3H, aromatic CH), 12.50 (s, 1H, NH); ^{13}C-NMR (101 MHz, DMSO-d_6): δ 28.9, 54.3, 60.0, 121.4, 124.9, 125.6, 126.3, 126.8, 127.9, 128.1, 131.5, 135.0, 149.2, 151.0, 152.4, 162.7 ppm. 2D ^{1}H-NMR NOESY spectra showed relevant NOE cross-peaks CH_2→aromatic CH. HRMS (ESI): m/z calculated for $C_{18}H_{16}N_4O_2$ $[M+H]^+$: 321.1307, found: 321.1363.

Enantioselective high-performance liquid chromatography analyses

The enantioseparation analyses were carried out with the Chirobiotic T column (250 x 4.6 mm I.D., containing the glycopeptide teicoplanin covalently bonded to a high purity 5 μm spherical silica gel), purchased from Sigma-Aldrich (Milano, Italy). Before the use, the column was conditioned with 40 mL of the selected mobile phase (HPLC-grade MeOH from Sigma-Aldrich). The HPLC measurements were made on a Shimadzu (Kyoto, Japan) Class-VP equipped with a EZ Start chromatography data software, a LC-10 ATVP pump, a SCL-10AVP system controller, a FCV-10ALVP low pressure gradient formation unit, a DGU-14A on-line degasser and a Rheodyne 7725i injector (Rheodyne, Cotati, CA, USA) with a 20 μL stainless steel loop. Column temperature was controlled through a Grace (Sedriano, Italy) heater/chiller (Model 7956R) thermostat. The chromatographic trace was obtained and handled with the LC Solution Software from Shimadzu (Kyoto, Japan). The employed eluent was degassed with 20 min sonication before use. The analyses were performed at a 1.0 mL/min eluent flow rate and with a column temperature fixed at 25°C.

The enantioseparation studies were performed according to a previous work by Beesley and Lee who reported an effective method for the enantioseparation of the 5-methyl-5-phenyl-hydantoin using a teicoplanin-based chiral stationary phase in a polar-organic (PO) mode of elution with neat

methanol as the eluent system[14]. The same chromatographic conditions were applied to evaluate the quality of the enantioselective synthetic protocols set up to obtain both intermediates **(-)-43** and **(+)-43**, and the optically active forms of racemic **(2)**. As a result, an enantiomeric excess values (e.e.) higher than 99% were obtained for both **(-)-43** and **(+)-43**. Afterwards, the chromatographic appraisal was carried out on the final products, providing for e.e. values higher than 93% and 96% for the (-)-**(*R*)-** and the (+)-**(*S*)**-isomer, (-)-**7** and (+)-**8**, respectively.

References

1. Willert, K. *et al.* Wnt proteins are lipid-modified and can act as stem cell growth factors. *Nature* **423,** 448–452 (2003).
2. Narwal, M., Haikarainen, T., Fallarero, A., Vuorela, P. M. & Lehtiö, L. Screening and structural analysis of flavones inhibiting tankyrases. *J. Med. Chem.* **56,** 3507–3517 (2013).
3. Kabsch, W. XDS. *Acta Crystallogr. D Biol. Crystallogr.* **66,** 125–132 (2010).
4. Vagin, A. & Teplyakov, A. Molecular replacement with MOLREP. *Acta Crystallogr. D Biol. Crystallogr.* **66,** 22–25 (2010).
5. Dodson, E. J., Winn, M. & Ralph, A. Collaborative Computational Project, number 4: providing programs for protein crystallography. *Meth. Enzymol.* **277,** 620–633 (1997).
6. Murshudov, G. N. *et al.* REFMAC5 for the refinement of macromolecular crystal structures. *Acta Crystallogr. D Biol. Crystallogr.* **67,** 355–367 (2011).
7. Emsley, P. & Cowtan, K. Coot: model-building tools for molecular graphics. *Acta Crystallogr. D Biol. Crystallogr.* **60,** 2126–2132 (2004).
8. Jones, G., Willett, P., Glen, R. C., Leach, A. R. & Taylor, R. Development and validation of a genetic algorithm for flexible docking. *J. Mol. Biol.* **267,** 727–748 (1997).
9. Thorsell, A.-G. *et al.* Structural Basis for Potency and Promiscuity in Poly(ADP-ribose) Polymerase (PARP) and Tankyrase Inhibitors. *J. Med. Chem.* **60,** 1262–1271 (2017).
10. Baumli, S., Hole, A. J., Noble, M. E. M. & Endicott, J. A. The CDK9 C-helix exhibits conformational plasticity that may explain the selectivity of CAN508. *ACS Chem. Biol.* **7,** 811–816 (2012).
11. Ndie, E., Cardinael, P., Schoofs, A. & Coquerel, G. An efficient access to the enantiomers of alpha-methyl-4-carboxyphenylglycine via a hydantoin route using a practical variant of preferencial crystallization AS3PC (auto seeded programmed polythermic preferental crystallization). *Tetrahedron: Asymmetry* **8,** 2913–2920 (1997).

12. Bucherer, H. . & Libe, V. . Syntheses of hydantoins. II. Formation of substituted hydantoins from aldehydes and ketones. *J. Prakt. Chem.* **141,** 5–43 (1934).

13. Coquerel, G., Petit, M. ., Bouaziz, R. & Depernet, D. Preparative resolution of some 5,5-disubstituted hydantoin derivatives via formation of diastereoisomeric salts. *Chirality* **4,** 400–403 (1992).

14. Beesley, T. E. & Lee, J. T. Method Development and Optimization of Enantioseparations Using Macrocyclic Glycopeptide Chiral Stationary Phases. in *Chiral Separation Techniques* (ed. Subramanian, G.) 1–28 (Wiley-VCH Verlag GmbH & Co. KGaA, 2006).
